# Use of Botulinum Toxin in Orofacial Clinical Practice

**DOI:** 10.3390/toxins12020112

**Published:** 2020-02-11

**Authors:** Maria-Angeles Serrera-Figallo, Gonzalo Ruiz-de-León-Hernández, Daniel Torres-Lagares, Alejandra Castro-Araya, Omar Torres-Ferrerosa, Esther Hernández-Pacheco, Jose-Luis Gutierrez-Perez

**Affiliations:** Oral Surgery Department, Dental School, University of Sevilla, 41009 Sevilla, Spain; ruizdeleong@gmail.com (G.R.-d.-L.-H.); alecastroaraya@yahoo.es (A.C.-A.); omartorres@gmail.com (O.T.-F.); estherhp@us.es (E.H.-P.); jlgp@us.es (J.-L.G.-P.)

**Keywords:** botulinum toxin, bruxism, salivary fistula, facial spasm, sialorrhea, orofacial dystonia, myofascial pain, facial paralysis, Frey syndrome, lockjaw, trigeminal neuralgia

## Abstract

Introduction: Botulinum neurotoxin (BoNT) is a potent biological toxin and powerful therapeutic tool for a growing number of clinical orofacial applications. BoNT relaxes striated muscle by inhibiting acetylcholine’s release from presynaptic nerve terminals, blocking the neuromuscular junction. It also has an antinociceptive effect on sensory nerve endings, where BoNT and acetylcholine are transported axonally to the central nervous system. In dentistry, controlled clinical trials have demonstrated BoNT’s efficiency in pathologies such as bruxism, facial paralysis, temporomandibular joint (TMJ) disorders, neuropathic pain, sialorrhea, dystonia and more. Aim: This study’s aim was to conduct a systematic literature review to assess the most recent high-level clinical evidence for BoNT’s efficacy and for various protocols (the toxin used, dilution, dosage and infiltration sites) used in several orofacial pathologies. Materials and methods: We systematically searched the MedLine database for research papers published from 2014 to 2019 with randomly allocated studies on humans. The search included the following pathologies: bruxism, dislocation of the TMJ, orofacial dystonia, myofascial pain, salivary gland disease, orofacial spasm, facial paralysis, sialorrhea, Frey syndrome and trigeminal neuralgia. Results: We found 228 articles, of which only 20 met the inclusion criteria: bruxism (four articles), orofacial dystonia (two articles), myofascial pain (one article), salivary gland disease (one article), orofacial spasm (two articles), facial paralysis (three articles), sialorrhea (four articles) or trigeminal neuralgia (three articles). Discussion: The clinical trials assessed showed variations in the dosage, application sites and musculature treated. Thus, applying BoNT can reduce symptoms related to motor muscular activity in the studied pathologies efficiently enough to satisfy patients. We did not identify the onset of any important side effects in the literature reviewed. We conclude that treatment with BoNT seems a safe and effective treatment for the reviewed pathologies.

## 1. Introduction

### 1.1. History

Botulinum toxin (Botox, BoNT) emerged in the 19th century when the Belgian bacteriologist van Ermengem discovered it in 1895. This bacterium produces a protein that can produce the most powerful neurotoxic substance known. In the 1920s, Sommer isolated BoNT type A (BoNT-A) in a purified form. In 1946, Schantz isolated the toxin in a crystalline form. In 1950, Brooks showed that the toxin blocks the release of acetylcholine in motor neurons, causing muscles to relax [1]. The earliest clinical application was described by Scott et al., who studied the possibility of a nonsurgical treatment for strabismus in primates in 1977. The first publication of its use as a therapeutic agent in humans occurred in 1980 [1].

In 2000, the Food and Drug Administration (FDA) approved Botox for treating cervical dystonia in adults to mitigate the abnormal head posture and neck pain associated with this condition. In 2002, the FDA approved Botox for treating moderate or severe glabellar wrinkles or frown lines in adult men and women. In 2004, it was approved to treat severe primary axillary hyperhidrosis not responding adequately to topical agents [2].

Regarding its mechanism of action, BoNTs enter nerve endings, cleaving and inactivating soluble N-ethylmaleimide-sensitive factor-attachment protein receptor (SNARE) proteins that are essential for releasing neurotransmitters. The protein’s nucleus contains heavy (100 kDa) and light (50 kDa) chains. The heavy chain helps internalize toxins, whereas the light chain, an endopeptidase, cleaves the SNARE complex. Clinically, the most important BoNT is type A, which cleaves protein 25, which is associated with the synaptosomal nerve (SNAP25). Type B, however, cleaves the VAMP (synaptobrevin) protein [3].

All BoNTs share a sustained action (three to six months), which makes them unique pharmacotherapeutic agents. Different BoNTs cleave different proteins within SNARE [4]. After their action time, new neuromuscular junctions and axonal connections are established that gradually replace the non-functional junctions. Muscle function is recovered after three to six months [5].

Interest in research on BoNT and in its clinical use has increased exponentially. This increase includes interest in the orofacial area, where BoNT is widely used for off-label treatments of several diseases arising from muscular hyperfunction and autonomic dysfunction.

### 1.2. Bruxism

This pathology is defined as repetitive masticatory activity characterized by clenching or grinding the teeth and/or thrusting the jaw. Bruxism may occur when awake or when asleep. Over time, this disorder causes tooth wear, temporomandibular joint (TMJ) and/or muscular pain, as well as joint locking and noise [6].

Current therapies for bruxism mainly minimize symptoms, reduce muscular activity with muscle relaxants, and protect dental and TMJ structures with splints [7].

BoNT is an efficient therapeutic option based on reducing activity in the muscles involved in this pathology (masseter, temporal and lateral pterygoid). BoNTs’ analgesic properties are also important for associated pain.

### 1.3. Dislocation of TMJ

This pathology is defined as the complete separation of the joint surfaces of the TMJ accompanied by symptoms such as pain, articular noises, a reduced mouth opening, muscular spasms and headache. This joint disorder’s etiology may relate to functional and neuromuscular TMJ problems, oral parafunctions, hereditary factors that coincide with ligament hyperelasticity, anatomical changes in joint surfaces and more [8].

Treatment for a dislocation of the TMJ is manual, using Nelaton’s maneuver. If chronic episodes of dislocation emerge, surgical intervention and infiltrations with BoNT are then applied [9].

### 1.4. Orofacial Dystonia

Dystonia is a neurological syndrome characterized by maintained and often repetitive involuntary muscle movements of antagonist muscles. Its etiology may be primary, of unknown cause, or secondary to neurological diseases, drugs or neoplasms [10].

### 1.5. Myofascial Pain

Myofascial pain syndrome may emerge in patients with a normal temporomandibular joint. Its etiological factors include: stress, tiredness or spasms in masticatory muscles (medial, internal, lateral or external pterigoids, both temporal and masseter). Symptoms include bruxism, spontaneous pain and palpation in and around the masticatory apparatus (or in other locations in the head and neck), and often abnormalities in mandibular mobility. A diagnosis is based on anamnesis and a physical examination. Conservative treatment is usually effective and includes analgesics, muscle relaxation, lifestyle changes and occlusal splints. [11].

Patients who do not respond to conservative treatment pose a therapeutic challenge for the clinician. The efficiency of intramuscular BoNT injections for chronic cases is still under investigation, but no standard treatment presently exists for refractory myofascial pain or for the concomitant arthralgia of the temporomandibular joint [12].

### 1.6. Salivary Cyst

Salivary gland lesions may be idiopathic, iatrogenic or post-traumatic and may result in a sialocele or a fistula. The majority of lesions return spontaneously, and BoNT A is one of several therapeutic possibilities [13].

### 1.7. Orofacial Spasm

Facial spasms are a complicated series of neurological motor disorders with unique diagnostic and treatment algorithms. Hemifacial spasm (HFS) is a chronic disease characterized by tonic and clonal involuntary contractions of muscles innervated by the ipsilateral facial nerve [14].

Injecting BoNT A is one of the most commonly used treatments for hemifacial spasms, given its high efficiency in controlling spasms. Despite its efficiency and low complication rate, the need for repeated injections is expensive and only relieves symptoms [15].

### 1.8. Facial Paralysis

Facial paralysis significantly impacts the quality of life. Patients suffer from functional deficiencies, cosmetic deformities, discomfort and social consequences. Chronic facial paralysis degenerates the involved facial muscles on the affected side, which makes the patient appear unduly aged. Contralateral facial hypertrophy also aggravates facial asymmetry [16].

Injecting BoNT-A is the cosmetic nonsurgical procedure with the highest rate of efficiency and satisfaction for patients. It combats spasms, synkinesis and the hyperactivity of skin muscles responsible for facial expressions. Although often used, no unified treatment protocol has been established [17].

### 1.9. Sialorrhea

Sialorrhea, or excessive salivary overflow, is a common and socially incapacitating symptom of many neurological disorders. Patients suffering from moderate to severe sialorrhea have a poor quality of life often worsened by complications such as aspiration pneumonia, oral infections, tooth decay and skin problems in the affected area [18].

Treating this pathology has two facets: the common treatment includes anticholinergic and antihistaminic pharmacotherapy (which is normally insufficient and often produces adverse effects) and surgical treatments to reduce saliva production. Although the results have been encouraging, no protocol has been established concerning the dosage and frequency of application [1].

### 1.10. Frey Syndrome

Frey syndrome was first described by Frey in 1923 as auricolotemporal syndrome—a postoperative complication of salivary gland surgery and, less frequently, of neck surgery, rhytidoplasty and trauma. It is characterized by sweating and gustatory flushing due to an aberrant reinnervation of the postganglionic parasympathetic neurons to the sweat glands and cutaneous blood vessels [19].

### 1.11. Trigeminal Neuralgia

Trigeminal neuralgia is one of the most incapacitating facial pain syndromes, and it significantly impacts quality of life. Compression of the trigeminal nerve may or may not affect its etiology.

Pharmacological treatment is based on anticonvulsant drugs, such as carbamazepine, whose drawback is reduced therapeutic efficiency over time, which necessitates surgical treatment to decompress the nerve. BoNT’s promising results could reveal a safe and effective therapeutic strategy for patients with drug-resistant idiopathic trigeminal neuralgia [20].

This review’s aim was to update the latest clinical use of this drug via recent randomized trials conducted in humans within the spectrum of the orofacial pathologies presented in this introduction.

## 2. Materials and Methods

A systematic search completed on 18 September 2019 in the Pubmed repertory identified the most up-to-date evidence available. The following searches were completed, each one focusing on pathologies we aimed to study: “botulinum toxin bruxism”; “botulinum toxin oromandibular dystonia”; “botulinum toxin facial spasm”; “botulinum toxin joint dislocation”; “botulinum toxin pain myofascial temporomandibular”; “botulinum toxin facial paralysis”; “botulinum toxin salivary cyst”; “botulinum toxin sialorrhea”; “botulinum toxin Frey syndrome” and “botulinum toxin trigeminal neuralgia.”

To identify the most up-to-date evidence and focus on use in humans, temporal (within the last five years) and content (in humans) filters were applied. The searches yielded the following results: “botulinum toxin bruxism” (25 articles); “botulinum toxin oromandibular dystonia” (31 articles); “botulinum toxin facial spasm” (48 articles); “botulinum toxin joint dislocation” (14 articles); “botulinum toxin pain myofascial temporomandibular” (12 articles); “botulinum toxin facial paralysis” (66 articles); “botulinum toxin salivary cyst” (13 articles); “botulinum toxin sialorrhea (73 articles)”; “botulinum toxin Frey syndrome (11 articles)” and “botulinum toxin trigeminal neuralgia” (39 articles).

Via journals at the University of Seville, we accessed the following articles from articles identified in the searches: “botulinum toxin bruxism” (17 articles); “botulinum toxin oromandibular dystonia” (24 articles); “botulinum toxin facial spasm” (39 articles); “botulinum toxin joint dislocation” (7 articles); “botulinum toxin pain myofascial temporomandibular” (12 articles); “botulinum toxin facial paralysis” (41 articles); “botulinum toxin salivary cyst” (13 articles); “botulinum toxin sialorrhea (50 articles)”; “botulinum toxin Frey syndrome (10 articles)” and “botulinum toxin trigeminal neuralgia” (25 articles).

Of these, only articles corresponding to randomized comparative studies were selected, excluding aside reviews and prospective and retrospective studies with no control group or randomization (Table 1).

Articles were selected from the following searches: “botulinum toxin bruxism” (4 articles); “botulinum toxin oromandibular dystonia” (2 articles); “botulinum toxin facial spasm” (2 articles); “botulinum toxin joint dislocation” (0 articles); “botulinum toxin pain myofascial temporomandibular” (1 articles); “botulinum toxin facial paralysis” (3 articles); “botulinum toxin salivary cyst” (1 articles); “botulinum toxin sialorrhea (4 articles)”; “botulinum toxin Frey syndrome (0 articles)” and “botulinum toxin trigeminal neuralgia” (3 articles).

## 3. Results

### 3.1. Bruxism

Adopting the inclusion criteria, the bibliographic review provided us with 10 articles, of which only four were randomized [21,22,23,24].

The first is a randomized 1:1 placebo-controlled parallel-design study with an open extension on 31 patients with nocturnal bruxism (13 patients with toxin infiltrations and 9 with placebos were chosen randomly). The participants were injected with 200 units of BoNT-A (60 units in each masseter and 40 in each temporal) or a placebo and were assessed at four and eight weeks after their initial treatment visit. The treatment’s effectiveness was measured using a visual analogue scale (VAS) for any change in bruxism and pain. In the BoNT-A group, sleep time increased and the bruxism episodes decreased. Two patients treated with BoNT-A presented episodes of smile deviation [21].

In another study, occlusal force and the therapeutic effectiveness of the masseter muscles were assessed after an intramuscular injection of BoNT A in patients with temporomandibular disorders (TMD) associated with bruxism. In this randomized study, 30 patients with TMD associated with bruxism were randomly allocated into three groups (*n* = 10 in each group) and treated with a bilateral intramuscular injection of BoNT-A in the masseter muscle, a placebo or a control. The occlusal force, bite duration, mouth closure, maximum occlusal force, and occlusal force distribution were measured and recorded. Occlusal force in the intercuspal position was reduced in the three groups. The authors observed a significant difference between the BoNT-A group and the placebo group [22].

In the next randomized study, the aim was to assess BoNT-A’s use with preoperative treatment via immediate-loading dental implants in patients with bruxism. The study included 26 patients (13 tests and 13 controls) with bruxism who were treated via extraction and subsequent implant placement in the extraction alveole. The mean follow-up time ranged from 18 to 51 months, with a mean time of 32.5 ± 10.4 months. In the test group, no problems with the implants were recorded. One patient presented bone loss of 1 to 2 mm around four of the placed implants; the other implants presented stable bone levels. In the control group, one patient lost two implants and another showed bone loss of 2 mm around three implants [23].

The final article described 24 patients divided randomly into three groups (*n* = 8) and treated using bilateral intramuscular injections of BoNT-A. Others were treated with a placebo, and the control group was administered no infiltrations. BoNT-A’s effect was assessed in the treatment of myofascial pain at rest and during chewing and occlusal force. The follow-up regimen occured at one week, three months and six months. Pain at rest improved in patients treated with BoNT. A significant change in maximum occlusal force was observed in the BoNT-A group in comparison with the other two groups [24].

To illustrate this treatment, we used the case of a 64-year-old, bruxomaniac patient with a 20-year evolution treated with splints and, in acute phases, with muscle relaxants, who presented with asymmetric masseter hypertrophy. In the previous year, the pain became more continuous, and treatment with Botox was proposed. The main advantages of this medicine for treating bruxism include: its administration in a single dose and individually for each patient increases its effectiveness and reduces side effects. It is not an exclusive therapy; conversely, it complements occlusal splints.

**Figure 1:**
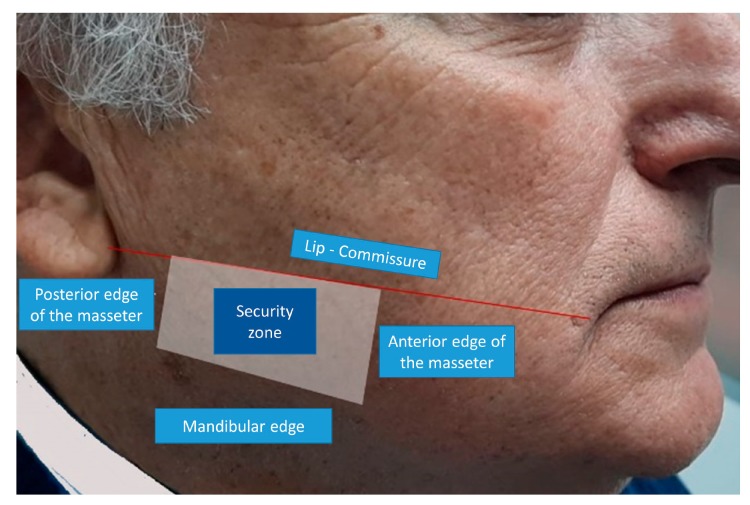
Safety limits for infiltration of the masseter: The upper limit is the commissure line to the earlobe (above the Stenon duct location). The anterior limit is the anterior edge of the masseter (the risorio muscle is in this area). The lower limit is the jaw’s lower edge. The posterior boundary is the masseter’s posterior border (the parotid gland).

The following treatment protocol was used: 125 units of Azzalure (Ipsen Biopharm Limited, Wrexham, UK), a BoNT-A *Clostridium botulinum*–hemagglutinin complex. The product was reconstituted with 1.25 mL of an injectable sodium chloride solution at 9 mg/mL (0.9%) in a graduated syringe in increments of 0.1 mL and 0.01 mL. This yielded a colorless reconstituted solution with a concentration of 10 U for 0.1 mL. The administration used a 1 mL syringe and a 30 G needle that was introduced perpendicular to the muscle’s thickness. The treatment was repeated at 20 weeks because administering injections at more frequent intervals or at higher doses may increase the risk of forming antibodies against the BoNT. Clinically, the formation of neutralizing antibodies may reduce the treatment’s effectiveness. The treatments should be spaced out until reaching a six-month interval between infiltrations (Figure 1).

### 3.2. Dislocation of TMJ

We did not find any randomized study for this pathology.

### 3.3. Orofacial Dystonia

In the orofacial dystonia bibliographic review, we found two randomized studies. The first was a prospective randomized study that revealed an improvement in quality of life in patients with orofacial dystonia with dysarthria receiving infiltration treatment using BoNT (the Quality of Communication Life Scale questionnaire of the American Speech-Language-Hearing Association was applied). In this study with 20 total patients (grouped as 10 control participants and 10 patients with oromandibular dystonia), dysarthria was assessed before and after treatment with the toxin. Significant differences were observed between the participating groups [25].

The second randomized study’s authors presented the management of oromandibular movement disorders associated with chorea acanthocytosis. Therapy was aimed at reducing severe oromandibular movements, dysphagia and mouth ulcers using electromyography-guided (EMG) BoNT treatments on masseter and lateral pterygoid muscles. This treatment minimized oral manifestations of this disease, majorly affecting patients’ quality of life because it improved chewing, swallowing and speech articulation [26].

### 3.4. Myofascial Pain

We found only one randomized clinical trial regarding this pathology. In the trial, the use of a low-level laser and the use of BoNT were compared in treating myofascial pain. The 15 total patients studied were randomly distributed in two groups. The laser group received a low-level GaAlAs (aluminium gallium arsenide) laser (100 mW of output power at a wavelength of 830 nm with continuous light emission), and the toxin group received 30 U of BoNT-A in the first session and 15 U 15 days later. The assessments were recorded using a VAS and a digital calibrator. Both investigated therapies effectively reduced pain, but the low-level laser’s effect was greater than that of BoNT-A. Neither treatment showed a statistically significant improvement in opening the mouth [27].

### 3.5. Salivary Cyst

In this study, we considered 13 articles regarding salivary cysts. Only one study was randomized. It was a phase 1, prospective, randomized, placebo-controlled and double-blind clinical trial investigating this treatment’s safety and effectiveness in preserving gland function after radiotherapy in 12 patients with head and neck cancer. BoNT-A, BoNT-B and a placebo were infiltrated. The time required for the treatment to take effect ranged from three to six weeks. No statistically significant differences were found between the two toxins. [28].

### 3.6. Orofacial Spasm

We found three articles with randomized studies in the facial spasm bibliographical review. The first article was a prospective, randomized and placebo-controlled study. Of the 44 total patients, two groups of patients with hemifacial spasm (HFS) were formed, one of 19 patients was treated with BoNT unilaterally, and the other 24 patients were treated bilaterally with BoNT-A. The BoNT-A doses on the affected side were standard doses. Facial asymmetry was studied using the Sunnybrook Facial Grading System, the Facial Evaluation Scale, the Symmetry Scale for Hemifacial Spasm (SSHS) and a self-assessment scale. The results showed improved facial asymmetry in patients treated bilaterally. The effects were stronger during voluntary facial movements than at rest. This therapy’s only drawback was increased treatment costs [29].

In this section’s penultimate study, the effectiveness of an ultrasound-guided BoNT injection in 157 patients with facial spasms was evaluated. These patients were divided randomly into two groups: oral treatments (78 cases) and BoNT-A treatment (79 cases). The therapeutic effect, duration, significant efficiency, and muscle spasm force were compared before and after treatment. The muscle spasm force was significantly lower in the second group after treatment (*p* < 0.01) [30].

To illustrate this type of treatment, we present a 26-year-old patient with a left hemifacial spasm that affected the major zygomatic muscle and minor zygomatic muscle and with an evolution of two years. This hemifacial spasm treatment’s main advantages include administration via a single dose and individually for each patient, which increases its effectiveness and decreases the side effects.

**Figure 2:**
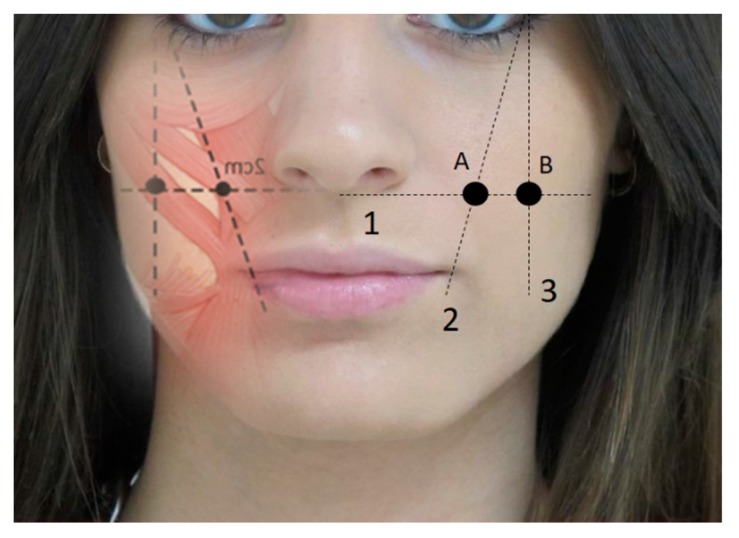
Guidelines for infiltrating major and minor zygomatic muscles. 1, line parallel to the nasal base; 2, line passing through the oral corner starting from the eye’s outer edge; 3, line perpendicular to one part of the eye’s outer edge. Infiltration points: (**A**) zygomatic minor point, (**B**) zygomatic major infiltration point. Dose per point = 2 U.

**Figure 3:**
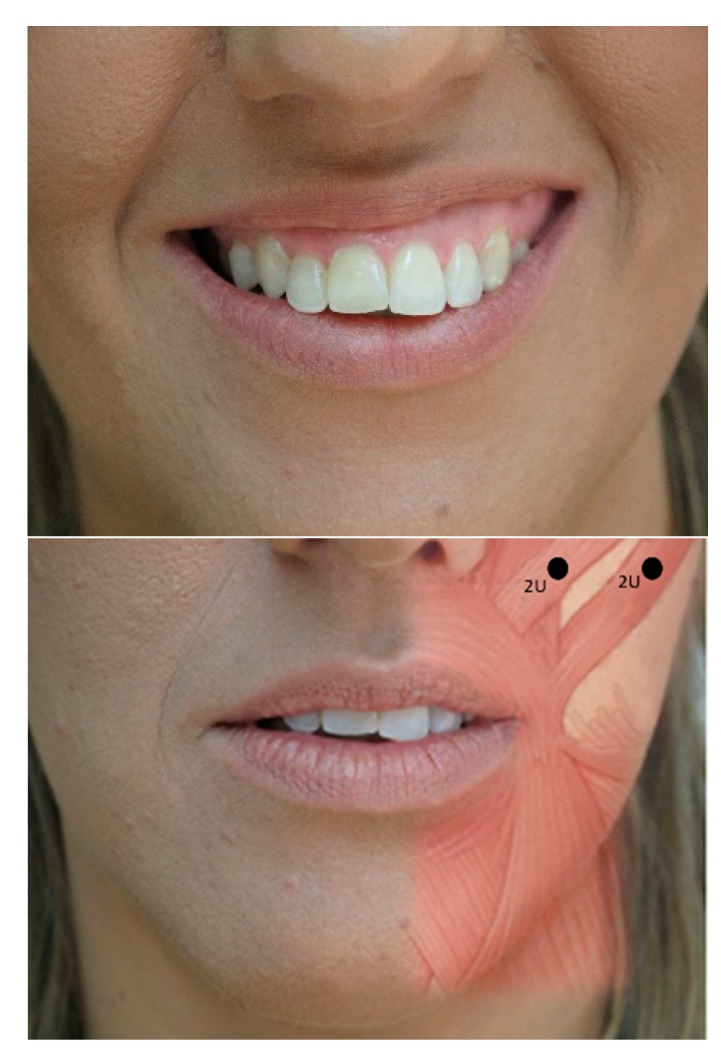
Evolution of spasm pre- (**top**) and post-treatment of two major and minor zygomatic points at 2 U per point (**bottom**).

The following treatment protocol was applied: 125 units of Azzalure, a BoNT-A of *Clostridium botulinum*–hemagglutinin complex. The product was reconstituted with 1.25 mL of an injectable sodium chloride solution of 9 mg/mL (0.9%) in a graduated syringe in increments of 0.1 and 0.01 mL, yielding a colorless reconstituted solution with a concentration of 10 U in 0.1 mL. It was administered using a 1 mL syringe and a 30 G needle introduced perpendicularly to the muscle’s thickness. The treatment was repeated at 16 weeks because administering injections at more frequent intervals or at higher doses may increase the risk of forming antibodies against the BoNT. Clinically, forming neutralizing antibodies can reduce the treatment’s effectiveness (Figure 2; Figure 3).

### 3.7. Facial Paralysis

In facial paralysis, the active side is treated with a toxin to achieve symmetry and balance. In the first study, the authors compared the conversion ratio (onabotulinum toxin A units to abobotulinum toxin A units) of 1:3, as they are not equivalents. A group of 55 patients with long-term facial paralysis were studied and treated randomly with injections of onabotulinum toxin A (*n* = 25) or abobotulinum toxin A (*n* = 30) on the non-paralyzed side. The follow-ups were performed at one and six months. The incidence of adverse effects was greater with abobotulinum toxin A. The treatment effect lasted six months, and the quality of life was similar in both groups [3].

In the next randomized study with 35 patients, researchers analyzed the analgesic effect of applying a cold gel pack (3–5 °C) for one minute using the VAS versus using a control gel pack (room temperature, 20 °C) before injecting the BoNT. The cold packs provided a statistically significant reduction in pain [31].

In this section’s final randomized study, a single-center, open and single-blind study, patients between 18 and 65 years old were recruited in the acute phase of facial nerve paralysis after surgical treatment of the posterior cranial fossa and cerebellopontine angle tumors. This study assessed the incobotulinum toxin’s effect in the acute and chronic phases of facial nerve paralysis after neurosurgical interventions. Patients received incobotulinum toxin A injections (the active treatment group) or standard rehabilitation treatment (the control group). The total dose of incobotulinum toxin was 40 to 50 U per patient, and the total number of injection sites varied from 10 to 15. Repeated injections were carried out four and eight months after starting the study. The active treatment group improved fastest, and synkinesis was lower in the toxin treatment group [32].

### 3.8. Sialorrhea

BoNT injections are commonly used to relieve severe drooling in patients with amyotrophic lateral sclerosis (ALS). In a prospective, randomized, and controlled pilot study, radiotherapy (*n* = 10; in the parotid and upper part of the submandibular gland) was compared with BoNT-A treatment (*n* = 10; only on the parotid gland due to the risk of increased oropharyngeal weakness) in patients with ALS. Between treatments, no significant differences in drooling were found. After the injections for patients with ALS, a dose of 200 U of BoNT-A was used for patients with lower motor neurons affected by adverse effects on oropharyngeal muscles [33].

This section’s second article was a single-center, randomized and controlled study to assess the effectiveness and safety of BoNT A injections in submandibular and parotid glands (as opposed to only in the parotid glands) to treat drooling in patients with spastic dyskinetic cerebral paralysis. The study also assessed the impact on quality of life. One group (group A) was treated with 100 units of BoNT-A, and the other group (group B) acted as a control. In the treatment group, all patients received a combined parotid and submandibular injection the first time, and then parotid-only injections. The authors found no significant advantage to injecting both submandibular and parotid glands over injecting only parotid glands [34].

BoNT is a therapeutic option for drooling in Parkinson’s disease, as identified by Narayanaswami et al. [35]. This study’s aim was to assess the effectiveness of incobotulinum toxin A for drooling in Parkinson’s disease via a randomized, double-blind, placebo-controlled crossover study. An incobotulinum toxin (100 units) or saline solution was injected into parotid (20 units) and submandibular glands (30 units). Subjects returned monthly for three assessments after each injection. For follow-up, they measured saliva weight, drooling frequency and severity. The authors reported no significant changes in the primary result for saliva weight one month after injection in the treatment period when compared with the placebo period [35].

In this section’s final article, researchers aimed to determine the most effective dose of BoNT-A to reduce sialorrhea in adults with neurological diseases. A prospective, randomized, double-blind and placebo-controlled trial was conducted for 24 weeks. Thirty patients with significant sialorrhea were randomly allocated to receive BoNT-A injections bilaterally in submandibular and parotid glands using an ultrasound guide. The total dose for BoNT-A patients was 50, 100 or 200 units. The primary result was the reduced saliva, measured by the differential weight (wet versus dry) of the intraoral dental dressing initially and at 2, 6, 12 and 24 weeks after the injection. The group that received 200 units of BoNT showed the greatest improvement [36].

### 3.9. Frey Syndrome

No randomized studies were found for this pathology.

### 3.10. Trigeminal Neuralgia

Zhang et al. conducted a randomized, double-blind, and placebo-controlled trial with a total of 84 patients who were treated with various doses of BoNT. Patients were randomly allocated to placebo (*n* = 28), 25 units of BoNT-A (*n* = 27), or 75 units of BoNT-A (*n* = 29). The results were analyzed using a VAS. The effect’s duration varied from one to eight weeks, and no significant VAS differences between the 25 units and 75 units groups were observed throughout the study. All adverse reactions were classified as minor or moderate [37].

In another study, two administration methods were compared: a single-dose strategy and a repeated-dose strategy. A total of 100 patients with classic symptoms of trigeminal neuralgia were recruited and distributed randomly and evenly into both groups. Patients in the single-dose group received a local injection of between 70 to 100 units of BoNT-A. The repeat-dose group received an initial injection of 50 to 70 units that was repeated after two weeks. All patients were monitored for six months. The groups were statistically similar in frequency, maximum time, VAS scores and adverse reaction rates. However, the effect duration increased significantly in the single-dose group [38].

Subcutaneous injections of BoNT-A are a promising treatment option for patients responding unsatisfactorily to pharmacological treatment or neurosurgical intervention. The effects last at least three months, so it could be a long-term treatment. This was the protocol for a clinical trial (with results not yet published) using a prospective, double-blind, placebo-controlled methodology to investigate a therapy to complement subcutaneous injections of BoNT-A for standard treatment in refractory classic trigeminal neuralgia [39].

## 4. Discussion

In our bibliographical review that addressed various pathologies in the orofacial area, we found few randomized studies that differed regarding the strain of toxin used, dosage, follow-up time or control scales. No standardized protocols have been published for any of the motor or sensory conditions studied, causing individualized and personalized treatment.

The bruxism articles highlight that, when a response to conservative treatment methods (such as the occlusal splint) is lacking, a BoNT may be an alternate effective treatment for nocturnal bruxism and chewing pain. Treatment with a BoNT appears beneficial in the case of bruxism, especially nocturnal bruxism, but several limiting factors, such as the high cost and need for repeated injections, prevent its widespread use [40].

Despite the small number of studies, BoNT-A seems a possible management option for sleep bruxism, minimizing symptoms and reducing the intensity of muscle contractions. Further studies are necessary, especially regarding treatment indications for bruxism itself [24].

The systematic literature review conducted by de la Torre to assess the effects of BoNT (BoNT-A) injections in bruxism treatment concludes that all studies that subjectively assessed jaw pain and stiffness showed positive results for treatment with BoNT-A. In contrast, the two studies with objective evaluations showed no reduction in bruxism episodes, but a decrease in the intensity of muscle contractions [7].

In one article, a study evaluated the safety and efficiency of onabotulinum toxin A (BoNT-A) injections for symptomatic sleep bruxism. None of the exploration elements changed significantly, but the total sleep time and the number and duration of bruxism episodes favored the BoNT-A group. Treatments with BoNT-A effectively and safely improved sleep bruxism in this placebo-controlled pilot trial. The authors advise a large multicenter test to confirm this encouraging data [21].

Another proposed treatment in this review is the preoperative use of BoNT in patients with bruxism who undergo immediate-loading prosthetic rehabilitation for implants. Given microanatomical changes that may advise against it, however, large-scale and long-term randomized clinical trials should be undertaken to determine additional benefits of this suggested treatment modality [23].

Oromandibular dystonia (OMD) is a focal dystonia involving the mouth, jaw and/or tongue that can be difficult to treat because many small muscles are involved. It can be classified as idiopathic, late or secondary dystonia or other neurological disorders, and it can be subdivided into opening the jaw, closing the jaw, deviation of the jaw and pursing of the lips [41].

BoNT is the treatment of choice in focal dystonia, but closure dystonia has a better response to treatment. However, those dystonias with greater complexity, such as opening or lingual dystonia, tend to have lower response rates [42].

Opening dystonia of the jaw is particularly difficult to treat, and extensively injecting the lateral pterygoid internally into a properly directed and submental muscle complex may be useful, but dysphagia can complicate this treatment. Using needle guides while injecting a BoNT into the lower head of the lateral pterygoid muscle helps to precisely and safely administer a BoNT for opening jaw dystonia by decreasing this adverse effect. Lingual dystonia is treated via infiltration of the hyoglossus and genioglossus muscles, but this should only be done when the disability warrants the risk of side effects. The complications include difficulty in chewing and dysphagia that can become severe, even with bronchoaspiration [26,43,44,45].

To reduce these adverse effects, start with the lowest possible dose then reassess the dose according to the patient’s response. Selecting the correct muscles to inject is of paramount importance, as is anatomical knowledge of the treated area [45].

The long-term DOF treatment may lose effectiveness in some patients due to several factors, such as the presence of antitoxin antibodies. Decreasing the toxin’s protein load prevents this effect. It is necessary to respect the treatment’s temporal guidelines to reduce overexposure. Another factor to consider is the disease’s evolution, which changes the involved muscle’s mechanical properties, making it more rigid and fibrous and decreasing the effectiveness of the treatment with TBA. The dose and the choice of muscle to infiltrate should be personalized in each session because dystonia’s intensity and location can change over time [46].

The pictures of late dyskinesias are clinically polymorphic with a reserved prognosis. They frequently cause disability, and no effective treatment currently exists. Most patients with tardive dyskinesia have a focal onset involving the cranio-cervical region. The oro-facio-lingual involvement is greater in tardive dyskinesia than in idiopathic dyskinesia. The most common form of DT is bucolingual dyskinesia. This DT is characterized by involuntary, uncoordinated and unapparent movements of the tongue that can vary from discretely excessive movements to hyperkinesia that interferes with speech, chewing and swallowing. Treatment with BTX administered by experienced neurologists has been an effective treatment in tardive dyskinesias. This treatment is particularly useful for patients with focal or segmental late dystonia and for late orolingual choreic movement. The toxin’s most relevant aspect is its safety and reversible adverse effects [47].

Salivary gland problems were also the subject of this bibliographical review. BoNT injections temporarily reduce saliva flow, so it is safe for treating salivary gland diseases. The main clinical side effect of BoNT injections is insufficiently reducing the saliva problem. In this case, infiltration should be repeated and with higher doses. It has also been used to treat recurrent parotitis because conservative methods may have limited effectiveness, and invasive approaches increase the risk of complications [48].

The glandular-level changes from treatment with BoNT are structural (a submandibular gland decrease) or biochemical (higher mucin, amylase and protein content) [49].

Treatments with BoNT-A or BoNT-B are clinically effective with appropriate doses. BoNT-B injections are more painful in most studies on muscle conditions, and the efficiency is shorter and the immunogenicity is higher. BoNT-B is used for sialorrhea, hyperhidrosis and other non-motor symptoms. In these condition, the efficiency of toxins A and B is comparable, and the dose ratio is 1:25–30 [50].

The safety and tolerability in treatments with BoNT A compared to B were evaluated based on patients’ personal reports. The effectiveness was evaluated via the benefit’s duration and via the Drooling Gravity Scale and the Drooling Frequency Scale four weeks after the intervention. The safety and efficiency of both toxins are comparable. Advanced age is significantly associated with a longer duration. Patients with Parkinson’s disease showed a more favorable safety–efficiency relationship than do patients with ALS, due to fewer adverse events and a longer treatment duration. None of this relates to the infiltrated toxin [51]. Onabotulinum toxin A and rimabotulinum toxin B injections are efficient and safe treatments for sialorrhea in patients with Parkinson’s disease [52]. In sialorrhea associated with ALS, serotypes A and B are considered effective and safe, even in the long term [53].

Another proposed therapy based on BoNT’s safety and effectiveness is the preservation of the glands’ functions after radiotherapy in patients with head and neck cancer. One study showed that BoNT can be safely combined with chemoradiotherapy. The dose and the time of the BoNT infiltration should be closely investigated for an effective analysis [25].

Some authors recommended, based on the doctor’s experience, a blind infiltration of BoNT in the parotid gland to treat salivary complications [54]. In the studies reviewed, an ultrasound guide was proposed to provide simple, real-time and non-invasive views of the salivary tissue adjacent to the sialoceles and fistulas. This could help infiltration in vessels and within the superficial lobe, which maximizes the drug’s effects on saliva production [18].

Treating recurrent TMJ dislocation with a BoNT was described above, and it was generally conducted using electromyographic monitoring for a precise injection into the muscle belly. Injecting BoNT A into the bilateral lateral pterygoid muscle indicated that the TMJ’s recurrent dislocation may be avoided for up to six months [55].

The majority of studies in our review reflect a BoNT’s significant benefits in treating patients with Frey syndrome. Due to a lack of evidence, we propose studies with adequate inclusion criteria and a controlled, randomized, multicenter approach with a high probability of high-quality evidence [56]. These studies should compare effective treatments (such as anticholinergic, antiperspirant, and BoNT) with control groups that use a placebo [57].

The quality of life is one of the most important variables when assessing satisfaction with treatment via BoNT for pathologies such as neuropathic pain, facial paralysis and synkinesis [20,40]. Treatment via BoNT-A is a safe and effective method for treating trigeminal neuralgia [40]. The reviewed studies showed this therapy can significantly alleviate pain and improve anxiety, depression and sleep, thus improving the quality of life. In some articles, the need for careful evaluation was mentioned for patients with neuropathic pain regarding their functional limitations and expectations for this treatment [20].

Treatment with a BoNT is particularly important when measuring the quality of life before and after various treatment modalities in patients with peripheral facial paralysis. Some authors reported that future studies should concern subjects with paralysis of a common etiology, and the quality of life should be validated using homogeneous instruments in all studies. Using a BoNT improved facial symmetry. Synkinesis, a secondary compensatory effect on the face’s non-paralyzed side or on the paralyzed side in spastic forms also benefits from this therapy. Treatment with a BoNT has the best results when complemented with rehabilitation and, in some cases, with surgical treatment [58]. In short, the treatment helps correct compensatory muscle hyperactivity and long-term synkinesis [33].

The BoNT is the treatment of choice for essential blepharospasm and hemifacial spasms, which has a similar effect than that obtained in synkinesis after facial nerve paralysis [59].

However, two negative factors must be considered. The first is that no standardized protocols exist in any of the studied motor or sensory conditions, causing individualized and personalized treatments. Treatment should be initiated with minimal doses that are increased depending on patient response. The second negative factor is this treatment’s cost and the need to administer it every three months. Despite the above and based on the articles reviewed, BoNT should be the option for patients who do not respond adequately to other oral therapies—not more invasive options.

The selected articles study some novel uses of BoNT concerning movement disorders, saliva secretion or neuropathic pain. Despite the lack of randomized controlled trials and the lack of FDA approval for these movement disorders, increasing evidence shows that BoNT benefits patients with these hyperkinetic movement disorders and that BoNT is a safe treatment when used by expert doctors [60].

The duration of a BoNT’s therapeutic effect is an important limitation. The treatment of chronic pathologies requires repeated treatments every three months for life, which annoys the patient and favors the emergence of resistance. Future studies should try to obtain therapeutic effects with longer durations.

## Figures and Tables

**Table 1:** Search results for articles regarding orofacial pathology included in the review.

Pathology	Articles				
	Review	Randomized	Prospective	Retrospective	Total
Bruxism	7	4	3	3	17
Dislocation of TMJ	3	-	4	-	7
Orofacial dystonia	10	2	9	3	24
Myofascial pain	7	1	1	3	12
Salivary gland disease	1	1	4	8	13
Orofacial spasm	18	2	11	7	39
Facial paralysis	16	3	9	13	41
Sialorrhea	17	4	11	18	50
Frey syndrome	7	-	1	2	10
Trigeminal neuralgia	14	3	8	-	25

## Abbreviations

BoNT	botulinum toxin
kDa	kilodalton
TMJ	temporomandibular joint
MD	myofascial disorders
HFS	hemifacial spasms
VAS	visual analogue scale
TMD	temporomandibular disorders

## References

[1] Carruthers A. (2003). History of the clinical use of botulinum toxin A and B. Clin. Dermatol..

[2] López del Val L.J., Castro García A. (2002). Toxina Botulínica: Aplicaciones Terapéuticas.

[3] do Nascimento Remigio A.F., Salles A.G., de Faria J.C., Ferreira M.C. (2015). Comparison of the efficiency of onabotulinumtoxinA and abobotulinumtoxinA at the 1:3 conversion ratio for the treatment of asymmetry after long-term facial paralysis. Plast. Reconstr. Surg..

[4] Galán Terraza A., Visa Nasarre J. (2012). Estado Actual del Tratamiento del Estrabismo.

[5] Tinastepe N., Küçük B.B., Oral K. (2015). Botulinum toxin for the treatment of bruxism. Cranio.

[6] Lobbezoo F., Ahlberg J., Raphael K.G., Wetselaar P., Glaros A.G., Kato T., Santiago V., Winocur E., De Laat A., De Leeuw R. (2018). International consensus on the evaluation of bruxism: Report of a work in progress. J. Oral Rehabil..

[7] De la Torre Canales G., Câmara-Souza M.B., do Amaral C.F., Garcia R.C., Manfredini D. (2017). Is there enough evidence to use botulinum toxin injections for bruxism management? A systematic literature review. Clin. Oral Investig..

[8] Oztel M., Bilski W.M., Bilski A. (2017). Botulinum toxin used to treat recurrent dislocation of the temporomandibular joint in a patient with osteoporosis. Br. J. Oral Maxillofac. Surg..

[9] Prechel U., Ottl P., Ahlers O.M., Neff A. (2018). The Treatment of Temporomandibular Joint Dislocation. Dtsch. Arztebl. Int..

[10] Jinnah H.A., Factor S.A. (2015). Diagnosis and treatment of dystonia. Neurol. Clin..

[11] Abboud W.A., Hassin-Baer S., Joachi M., Givol N., Yahalom R. (2017). Localized myofascial pain responds better than referring myofascial pain to botulinum toxin injections. Int. J. Oral Maxillofac. Surg..

[12] Thomas N.J., Aronovich S. (2017). Does Adjunctive Botulinum Toxin a Reduce Pain Scores When Combined with Temporomandibular Joint Arthroscopy for the Treatment of Concomitant Temporomandibular Joint Arthralgia and Myofascial Pain?. J. Oral Maxillofac. Surg..

[13] Steffen A., Hasselbacher K., Heinrichs S., Wollenberg B. (2014). Botulinum Toxin for Salivary Disorders in the Treatment of Head and Neck Cancer. Anticancer Res..

[14] Yuksel B., Genc F., Yaman A., Goksu E.O., Ak P.D., Gomceli Y.B. (2019). Evaluation of stigmatization in hemifacial spasm and quality of life before and after botulinum toxin treatment. Acta Neurol. Belg..

[15] Xiao L., Pan Y., Zhang X., Hu Y., Cai L., Nie Z., Pan L., Li B., He Y. (2016). Facial asymmetry in patients with hemifacial spasm before and after botulinum toxin A treatment. Neurol. Sci..

[16] Choe W.J., Kim H.D., Han B.H., Kim J. (2017). Thread lifting: A minimally invasive surgical technique for long-standing facial paralysis. HNO.

[17] Sundaram H., Signorini M., Lie S., de Almeid A.R.T., Wu Y., Vieira Bra A., Fagien S., Goodman G.J., Monheit G., Raspaldo H. (2016). Global Aesthetics Consensus: Botulinum Toxin Type A-Evidence-Based Review, Emerging Concepts, and Consensus Recommendations for Aesthetic Use, Including Updates on Complications.; Global Aesthetics Consensus Group. Plast. Reconstr. Surg..

[18] Barbero P., Busso M., Artusi C.A., De Mercanti  S., Tinivella M., Veltri A., Durelli L., Clerico M.J. (2016). Ultrasound-guided Botulinum Toxin-A Injections: A Method of Treating Sialorrhea. J. Vis. Exp..

[19] Motz K.M., Kim Y.J. (2016). Auriculotemporal Syndrome (Frey Syndrome). Otolaryngol. Clin. N. Am..

[20] Park J., Park H.J. (2017). Botulinum Toxin for the Treatment of Neuropathic Pain. Toxins.

[21] Ondo W.G., Simmons J.H., Shahid M.H., Hashem V., Hunter C., Jankovic J. (2018). Onabotulinum toxin-A injections for sleep bruxism: A double-blind, placebo-controlled study. Neurology.

[22] Zhang L.D., Liu Q., Zou D.R., Yu L.F. (2016). Occlusal force characteristics of masseteric muscles after intramuscular injection of botulinum toxin A (BTX—A) for treatment of temporomandibular disorder. Br. J. Oral Maxillofac. Surg..

[23] Mijiritsky E., Mortellaro C., Rudberg O., Fahn M., Basegmez C., Levin L. (2016). Botulinum Toxin Type A as Preoperative Treatment forImmediately Loaded Dental Implants Placed in Fresh Extraction Sockets for Full-Arch Restoration of Patients with Bruxism. J. Craniofac. Surg..

[24] Jadhao V.A., Lokhande N., Habbu S.G., Sewane S., Dongare S., Goyal N. (2017). Efficacy of botulinum toxin in treating myofascial pain and occlusal force characteristics of masticatory muscles in bruxism. Indian J. Dent. Res..

[25] Page A.D., Siegel L., Jog M. (2017). Self-Rated Communication-Related Quality of Life of Individuals with Oromandibular Dystonia Receiving Botulinum Toxin Injections. Am. J. Speech Lang. Pathol..

[26] Ortega M.C., Skármeta N.P., Diaz Y. (2016). Management of oromandibular dystonia on a chorea acanthocytosis: A brief review of the literature and a clinical case. J. Cranio.

[27] De Carli B.M., Magro A.K., Souza-Silva B.N., de Souza Matos F., De Carli J.P., Paranhos L.R., Magro E.D. (2016). The effect of laser and botulinum toxin in the treatment of myofascial pain and mouth opening: A randomized clinical trial. J. Photochem. Photobiol. B.

[28] Teymoortash A., Pfestroff A., Wittig A., Franke N., Hoch S., Harnisch S., Schade-Brittinger C., Hoeffken H., Engenhart-Cabillic R., Brugger M. (2016). Safety and Efficacy of Botulinum Toxin to Preserve Gland Function after Radiotherapy in Patients with Head and Neck Cancer: A Prospective, Randomized, Placebo-Controlled, Double-Blinded Phase I Clinical Trial. PLoS ONE.

[29] Xiao L., Pan L., Li B., Zhou Y., Pan Y., Zhang X., Hu Y., Dressler D., Jin L. (2018). Botulinum toxin therapy of hemifacial spasm: Bilateral injections can reduce facial asymmetry. J. Neurol..

[30] Ding X.D., Chen H.X., Xiao H.Q., Wang W., Wang H., Zhang G.B. (2015). Efficiency of ultrasound and water capsule-guided local injection of botulinum toxin type A treatment on patients with facial spasm. Eur. Rev. Med. Pharmacol. Sci..

[31] Pucks N., Thomas A., Hallam M.J., Venables V., Neville C., Nduka C. (2015). Cutaneous cooling to manage botulinum toxin injection-associated pain in patients with facial palsy: A randomized controlled trial. J. Plast. Reconstr. Aesthet. Surg..

[32] Akulov M.A., Orlova O.R., Orlova A.S., Usachev D.J., Shimansky V.N., Tanjashin S.V., Khatkova S.E., Yunosha-Shanyavskaya A.V. (2017). IncobotulinumtoxinA treatment of facial nerve palsy after neurosurgery. J. Neurol. Sci..

[33] Weikamp J.G., Schinagl D.A., Verstappen C.C., Schelhaas H.J., de Swart B.J., Kalf J.G. (2016). Botulinum toxin-A injections vs radiotherapy for drooling in ALS. Acta Neurol. Scand..

[34] Gonzalez-L M.D., Martinez C., Bori Y., Fortuny I., Suso-Vergara S. (2017). Factors in the Efficacy, Safety, and Impact on Quality of Life for Treatment of Drooling with Botulinum Toxin Type A in Patients with Cerebral Palsy. Am. J. Phys. Med. Rehabil..

[35] Narayanaswami P., Geisbush T., Tarulli A., Raynor E., Gautam S., Tarsy D., Gronseth G. (2016). Drooling in Parkinson’s disease: A randomized controlled trial of incobotulinum toxin A and meta-analysis of Botulinum toxins. Parkinsonism Relat. Disord..

[36] Mazlan M., Rajasegaran S., Engkasa J.P., Nawawi O., Goh K.J., Freddy S.J. (2015). A Double-Blind Randomized Controlled Trial Investigating the Most Efficacious Dose of Botulinum Toxin-A for Sialorrhea Treatment in Asian Adults with Neurological Diseases. Toxins.

[37] Zhang H., Lian Y., Ma Y., Chen Y., He C., Xie N., Wu C. (2014). Two doses of botulinum toxin type A for the treatment of trigeminal neuralgia: Observation of therapeutic effect from a randomized, double-blind, placebo-controlled trial. J. Headache Pain.

[38] Zhang H., Lian Y., Xie N., Chen C., Zheng Y. (2017). Single-dose botulinum toxin type a compared with repeated-dose for treatment of trigeminal neuralgia: A pilot study. J. Headache Pain.

[39] Burmeister J., Holle D., Bock E., Ose C., Diener H.C., Obermann M. (2015). Botulinum neurotoxin type A in the treatment of classical Trigeminal Neuralgia (BoTN): Study protocol for a randomized controlled trial. Trials.

[40] Asutay F., Atalay Y., Asutay H., Acar A.H. (2017). The Evaluation of the Clinical Effects of Botulinum Toxin on Nocturnal Bruxism. Pain Res. Manag..

[41] Comella C.L. (2018). Systematic review of botulinum toxin treatment for oromandibular dystonia. Toxicon.

[42] Moscovich M., Chen ZP., Rodriguez R. (2015). Successful treatment of open jaw and jaw deviation dystonia with botulinum toxin using a simple intraoral approach. J. Clin. Neurosci..

[43] Nastasi L., Mostile G., Nicoletti A., Zappia M., Reggio E., Catania S. (2016). Effect of botulinum toxin treatment on quality of life in patients with isolated lingual dystonia and oromandibular dystonia affecting the tongue. J. Neurol..

[44] Yoshida K. (2018). Computer m Toxin into the Lateral Pterygoid Muscle in Patients with Oromandibular Dystonia. Pain Res. Manag..

[45] Tan EK., Jankovic J. (1999). Botulinum toxin A in patients with oro–mandibular dystonia. Long–term follow–up. Neurology.

[46] Ramirez-Castaneda J., Jankovic J. (2014). Long-term efficacy, safety, and side effect profile of botulinum toxin in dystonia: A 20-year follow-up. Toxicon.

[47] Vijayakumar D., Jankovic J. (2016). Drug-Induced Dyskinesia, Part 2: Treatment of Tardive Dyskinesia. Drugs.

[48] O’Neil L.M., Palme C.E., Riffat F., Mahant N. (2016). Botulinum Toxin for the Management of Sjögren Syndrome-Associated Recurrent Parotitis. J. Oral Maxillofac. Surg..

[49] Cisneros-Lesser J.C., Sabas Hernández-Palestina M. (2017). Treatment of patients with sialorrhea. A systematic review. Investig. Discapac..

[50] Bentivoglio A.R., Del Grande A., Petracca M., Ialongo T., Ricciard L. (2015). Clinical differences between botulinum neurotoxin type A and B. Toxicon.

[51] Petracca M., Guidubaldi A., Ricciardi L., Ialongo T., Del Grande A., Mulas D., Di Stasio E., Bentivoglio A.R. (2015). Botulinum Toxin A and B in sialorrhea: Long-term data and literature overview. Toxicon.

[52] Cardoso F. (2018). Botulinum toxin in parkinsonism: The when, how, and which for botulinum toxin injections. Toxicon.

[53] Banfi P., Ticozzi N., Lax A., Guidugli G.A., Nicolini A., Silani V. (2015). A review of options for treating sialorrhea in amyotrophic lateral sclerosis. Respir. Care.

[54] Tighe D., Williams M., Howett D. (2015). Treatment of iatrogenic sialoceles and fistulas in the parotid gland with ultrasound-guided injection of botulinum toxin A. Br. J. Oral Maxillofac. Surg..

[55] Tocaciu S., McCullough M.J., Dimitroulis G. (2019). Surgical management of recurrent TMJ dislocation-a systematic review. J. Oral Maxillofac. Surg..

[56] Xie S., Wang K., Xu T., Guo X.S., Shan X.F., Cai Z.G. (2015). Efficacy and safety of botulinum toxin type A for treatment of Frey’s syndrome: Evidence from 22 published articles. Cancer Med..

[57] Li C., Wu F., Zhang Q., Gao Q., Shi Z., Li L. (2015). Interventions for the treatment of Frey’s syndrome. Cochrane Database Syst. Rev..

[58] Benichou L., Labbe D., Le Louarn C., Guerreschi P. (2015). Séquelles de paralysie faciale et toxine botulique Facial palsy sequel and botulinum toxin. Ann. Chir. Plast. Esthét..

[59] Wabbels B. (2018). Botulinum Toxin—New Developments in Ophthalmology. Klin. Mon. Augenheilkd..

[60] Jankovic J. (2018). An update on new and unique uses of botulinum toxin in movement disorders. Toxicon.

